# Associations between mindfulness and non-restorative sleep: the roles of resilience and handgrip

**DOI:** 10.3389/fpsyg.2024.1476197

**Published:** 2025-01-07

**Authors:** Shuhan Li, Yingting Jiang, Zhenrong Shen, Yuan Liao, Yihao Zeng, Zengjie Ye

**Affiliations:** ^1^School of Nursing, Guangzhou Medical University, Guangzhou, China; ^2^School of Nursing, Guangzhou University of Chinese Medicine, Guangzhou, China

**Keywords:** mindfulness, resilience, non-restorative sleep, handgrip, moderated mediation analysis, generalized additive model analysis

## Abstract

**Aim:**

This study examined the mediating role of resilience and the moderating role of grip strength (handgrip) in the relationship between mindfulness and non-restorative sleep (NRS) and evaluated the non-linear association between mindfulness and NRS among university freshmen students.

**Design:**

This is a cross-sectional descriptive study.

**Method:**

A total of 384 university students were recruited from Be Resilient to Nursing Career (BRNC) from two universities in June 2022. The Mindful Attention Awareness Scale, 10-item Connor–Davidson Resilience Scale, Non-restorative Sleep Scale, and handgrip were administered. Latent profile analysis, moderated mediation analysis, and generalized additive model analysis were performed.

**Results:**

The following three subgroups were identified through the latent profile analysis: low mindfulness (21%), medium mindfulness (49.4%), and high mindfulness (29.6%). While the significant mediating role of resilience between mindfulness and NRS was confirmed (SE = 0.041, *p* < 0.05), the moderating role of handgrip was not statistically significant. A non-linear relationship was verified between mindfulness and NRS.

## Introduction

Adolescents are often exposed to environmental and health threats during their rapid physical and mental development (Han et al., 2023), including sleep disorders and stress (Bodys-Cupak et al., 2022; Minshew et al., 2023). Non-restorative sleep (NRS), a product of sleep disorders, can cause fatigue and poor concentration, and can negatively affect daily activities and physical health (Edinger et al., 2004). It is strongly associated with conditions such as personal environmental safety, somatic health, anxiety, and depression (Chiu et al., 2014; Li et al., 2021a; Sarsour et al., 2010). Therefore, it is necessary to identify groups experiencing NRS and implement necessary interventions to improve the physical and mental health of university freshmen students and promote their academic development.

Mindfulness was originally considered an intervention that refers to the consciousness-directed focusing of an individual’s attention on experiencing the present moment through meditation (Creswell et al., 2019; Kabat-Zinn and Living, 1990). As research progressed, mindfulness came to be seen as a trait or a stable internal character of individuals (Rapgay and Bystrisky, 2009). It helps individuals to effectively cope with stress, anxiety, and other negative emotions; promotes positive emotions; and improves sleep quality by consciously being aware of and controlling the autonomic nervous system to strengthen self-regulation (Lengacher et al., 2016; Shapiro et al., 2006). Currently, mindfulness has been shown to have a dominant correlation with insomnia, high mindfulness level which helps improve individuals’ sleep quality (Wen et al., 2022). The International Classification of Sleep Disorders defined NRS as one of the sub-types of primary insomnia (Vernon et al., 2010), unlike other typical insomnia symptoms, it presented symptoms of daytime sleepiness and other daytime consequences. Despite the etiology of NRS remains unknown, previous research has confirmed the closely correlation between NRS and sleep–wake factors (Zhang et al., 2012). Mindfulness exercise as a good entry point to intervene in sleep disorders, can promote experiential awareness of a range of experiences by training sustained attention and attention inhibition in the primary arousal stage. And learning to accept and adjust their relationship to their experience in the secondary arousal and distorted perceptions stages, in order to accept negative perceived thoughts and physical sensations so as to reach the purpose of improve sleep–wake status. In other word, mindfulness can target and positively affect each of the cognitive and behavioral vulnerabilities related to sleep disorder symptoms (Shallcross et al., 2019). However, the relationship between mindfulness and NRS remains under-explored. Therefore, it is necessary to further explore internal waves to enhance accuracy.

Resilience is the ability of an individual to maintain a positive mindset in the face of adversity, stress, and challenge, and to recover and adapt quickly to change (Lazarus, 1993). Mindfulness positively predicts resilience (Bajaj et al., 2022; Zhang et al., 2023). Kwak et al. (2019) reported that meditative interventions help enhance the functional connectivity of the neocortex during resting states to promote resilience in individuals and neurologically confirm the underlying mechanism by which positive thinking affects mental toughness. Additionally, resilience may have important implications for NRS. University freshmen students’ failure to cope with hindrances owing to interference from factors such as their own inability or social avoidance eventually leads to a buildup of negative emotions that affect their sleep status (Almojali et al., 2017; Alotaibi et al., 2020). Resilience is an internal coping resource that helps freshmen students adjust their emotions and cognitive judgments better to reduce the impact of failure (Yan and Gai, 2022). Groups with strong resilience tend to avoid sleep disturbances and make sleep adjustments (Colrain, 2010). Therefore, the present study aimed to investigate the mediating role of mental toughness in the relationship between mindfulness and NRS.

In addition, physical inactivity has been suggested to be a predictor of NRS incidence (Li et al., 2021b; Otsuka et al., 2023). Grip strength (handgrip) is an irreplaceable indicator of whole-body skeletal muscle function (Thangavel et al., 2014). It is the strength produced by the largest muscle group in the hands (Lee, 2021). To some extent, the level of grip strength is a reflection of the health of the body (Son et al., 2018). Grip strength is also an independent predictor of sleep quality, and people with better handgrip tend to have better sleep quality and are less likely to suffer from sleep disorders (Ling Ling et al., 2019; Pana et al., 2021). Relationships between mindfulness and handgrip has been found by previous research. Mindfulness had a immediate effect on muscular fitness (Dueland, 2023). However, it remains unknown whether there was a moderate effect of handgrip on correlation between mindfulness and NRS. Therefore, exploring handgrip can help to further understand ways to reduce individual NRS.

In summary, although mindfulness, resilience, and handgrip may be important factors affecting NRS (Colrain, 2010; Na et al., 2022; Otsuka et al., 2023), the association between the four has not fully been discussed. Thus, the present study proposed the following hypotheses:

*H1*: Heterogeneity exists in mindfulness among university freshmen students.

*H2*: Resilience plays a mediating role between mindfulness and NRS.

*H3*: Handgrip moderates the relationship between mindfulness and NRS.

*H4*: There is a positive non-linear relationship between mindfulness and NRS.

## Method

### Design and participants

The present study was conducted with 384 freshmen students from two universities in May 2022. The study criteria were met as follows: (1) voluntary participation, (2) ability to write correctly in Chinese, and (3) newly enrolled university freshmen students in 2021. Those with major mental disorders or illnesses were excluded. An initial review of the data revealed that 14 (3.6%) questionnaires had a large number of missing values. These were excluded from the study, resulting in a final sample of 370 (response rate = 96.4%).

### Sample size

A minimal sample size of about 300 lends enough accuracy in identifying correct profiles (Kim, 2014). Thus, 370 was efficiently powerful for the latent profile analysis (LPA) in this study.

### Ethical approval

This study was approved by Be Resilient to Nursing Career (BRNC). Other details can be obtained from previous studies (Li et al., 2023; Mei X. et al., 2022; Mei et al., 2022a; Mei et al., 2022b). Before the study, an informed consent form was signed by the participants. The individual information of the respondents was kept strictly confidential.

## Instrument

### Demographic characteristics

Based on relevant studies (Bamber and Schneider, 2022; Moeller et al., 2020; Vidic and Cherup, 2019), demographic characteristics such as gender, major, parental marital status, and whether parents live separately were contained.

### Mindful attention awareness scale

The Mindful Attention Awareness Scale (MAAS), developed by Brown and Ryan (2003), assesses individual differences in mindful state. Chinese version was developed by (Deng et al., 2012). The MAAS includes 15 items, and the score ranges from 0 to 90, and higher scores mean higher current awareness and attention traits, which has been proven to be a one-factor structure. The scale has been broadly utilized among freshmen students (Gao et al., 2016; Shi et al., 2018). The Cronbach’s *α* in this study was 0.846.

### 10-item Connor–Davidson resilience scale

The 10-item Connor–Davidson Resilience Scale (CD-RISC-10) was developed by Campbell-Sills and Stein (2007), and the Chinese version of the scale was evaluated by Ye et al. (2017) and its single dimension was retained. Its score ranges from 0 to 40, with a higher score reflecting a higher level of resilience. The validity and reliability of the Chinese version of CD-RISC-10 have been widely verified among college students (Cheng et al., 2020; Notario-Pacheco et al., 2011). Cronbach’s α in this study was 0.879.

### Non-restorative sleep scale

The Non-restorative Sleep Scale (NRSS), developed by Wilkinson and Shapiro (2013), consists of the following four dimensions: refreshment from sleep, physical or medical symptoms of NRS, daytime functioning, and affective symptoms. The Chinese version of NRSS has been proven (Li et al., 2019), and its score ranges from 12 to 60, with a higher score indicating lower NRS. The Cronbach’s α in this study was 0.789.

### Handgrip strength

Handgrip strength was assessed using an electric hand dynamometer (CAMRY; 90 kg/198 lb). As per the Chinese National Physical Fitness Measurement Standards (Feng et al., 2021), participants were required to be in a standing position, with their arms naturally drooping. On a verbal cue, participants had to firmly use their dominant hand to squeeze the dynamometer and hold for 5 s. The action had to be repeated after a rest period of at least 1 min. The average value of two results was recorded in this study.

### Data analysis

Before data analysis, descriptive statistics were examined for the variables as mean (SD) and proportions (%). Then, univariate analysis was performed to determine the underlying factors affecting NRS. Pearson’s analysis was performed to evaluate the correlations among mindfulness, resilience, NRS, and handgrip. The strength of relationships was categorized as follows (Sedgwick, 2012): weak (*r* < 0.3), moderate (0.3 ≤ *r*<0.5), and strong (*r* ≥ 0.5). Second, based on the MAAS scores, an LPA was conducted to identify potential subgroups of mindfulness. We adhered to the indicators of Lo–Mendell–Rubin adjusted likelihood ratio test (LMR), and entropy as references (Tein et al., 2013). Furthermore, potential factors for different LPA-based profiles were identified via a univariate logistic regression. A Bayesian independent sample *t*-test was performed to compare NRS based on LPA profiles. Third, Harman’s single factor test was conducted to evaluate the potential existence of the common method bias. A mediation analysis was applied to validate the mediating role of resilience in the relationship between mindfulness and NRS. Fourth, a moderation analysis was utilized to verify the moderating role of handgrip between mindfulness and NRS. Fifth, a generalized additive model analysis was performed to examine whether there is a non-linear fitting relationship between mindfulness and NRS. Statistical analyses were conducted using SPSS Version 26.0 (Armonk, NY: IBM Corp), Mplus (version 8.3), JASP (0.16.1), R (X64 4.1.1), and Empower Stats (version 4.1).

## Results

### Demographic characteristics

**Figure 1:**
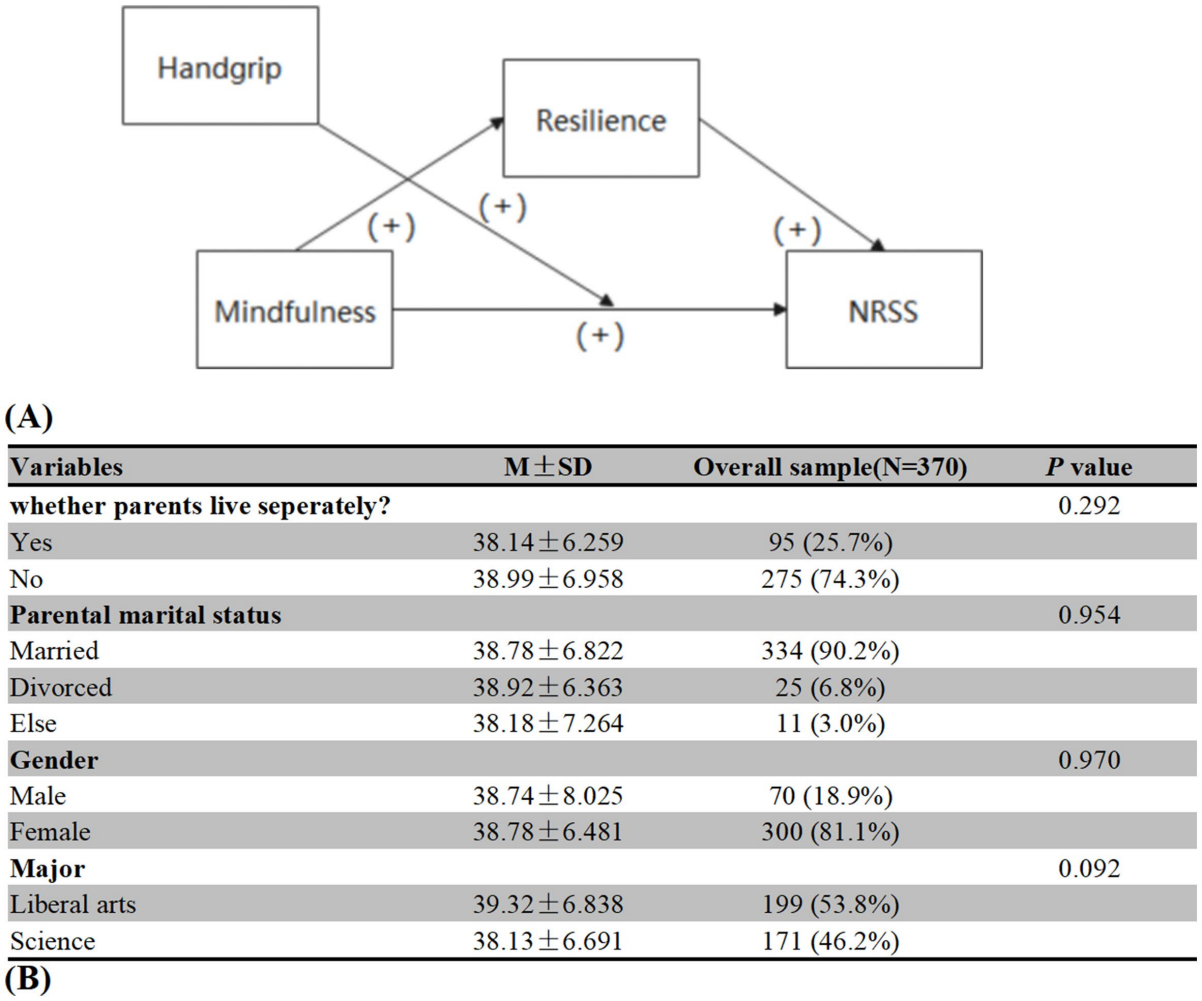
Conceptual model and demographic analysis among freshmen students.

Of the total number of participants, 81.1% were female and 72.7% were liberal arts majors. Demographic characteristic showed no significant differences in our study. Figure 1 provides other details.

### Latent profile analysis of mindfulness

**Figure 2:**
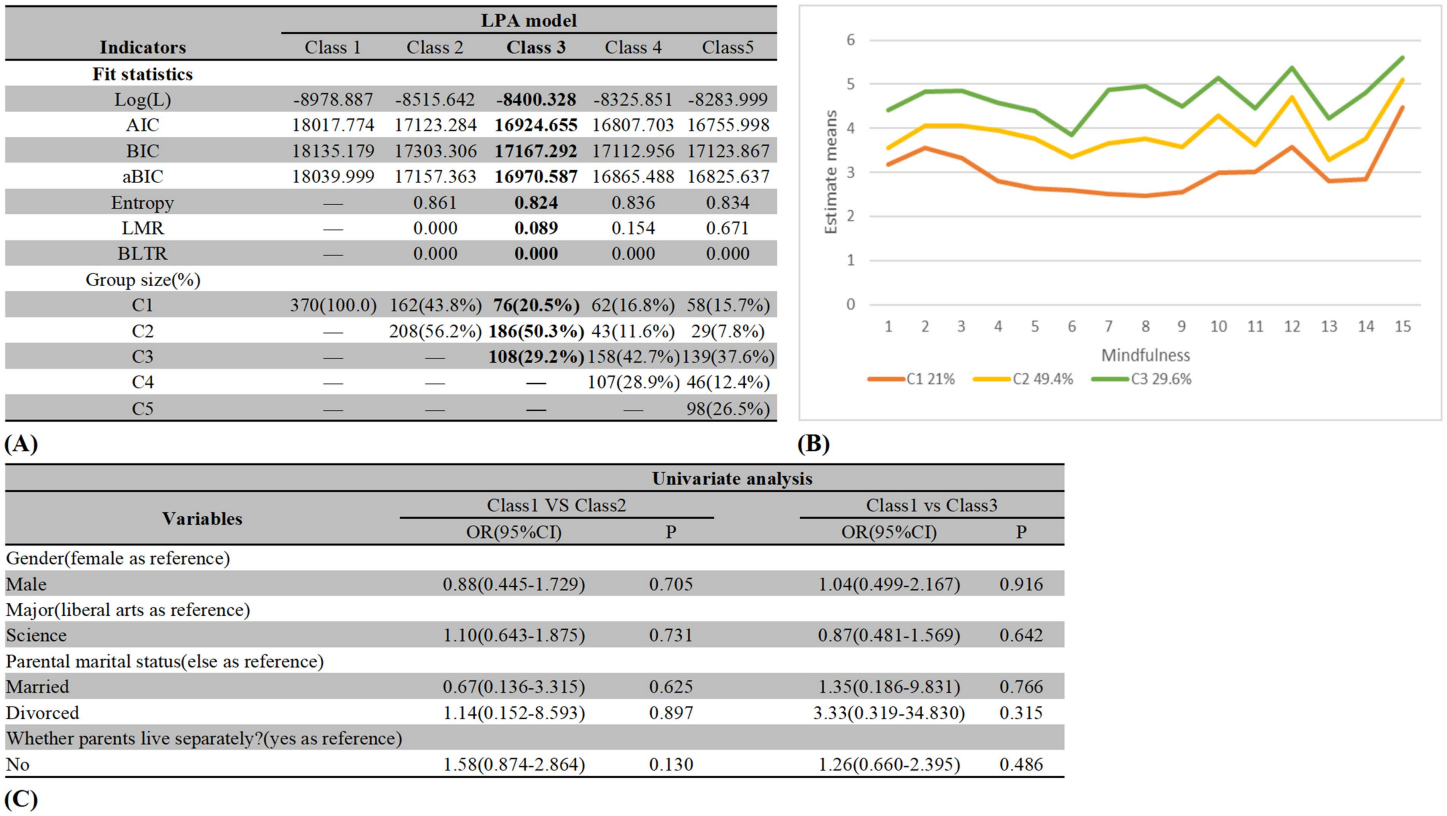
Fitting index and group size of latent profile analysis models and logistic regression results based on LPA.

Figure 2A shows the fit indicators of the different LPA models. The LMR was not significant for three-class, four-class, and five-class models. Furthermore, AIC, BIC, and aBIC tended to decrease with an increase in the number of classes. Thus, data alone cannot be used to select optimal profiles. Although the two-class model had acceptable LMR *p* values (*p* < 0.01), the class probability was too simple to response clinical traits well. The four-class model had a low proportion of class probability (Class 2 = 11.6%, *N* = 42). Overall, the three-class model was suitable for our study. The parameters of the three-class model were termed low mindfulness (class1, 20.5%), medium mindfulness (class2, 50.3%), and high mindfulness (class3, 29.2%). The univariate analysis showed no significant indicator to profile types. Figures 2B,C provides other details.

### Latent profile analysis-based difference in nonrestorative sleep scale scores

**Figure 3:**
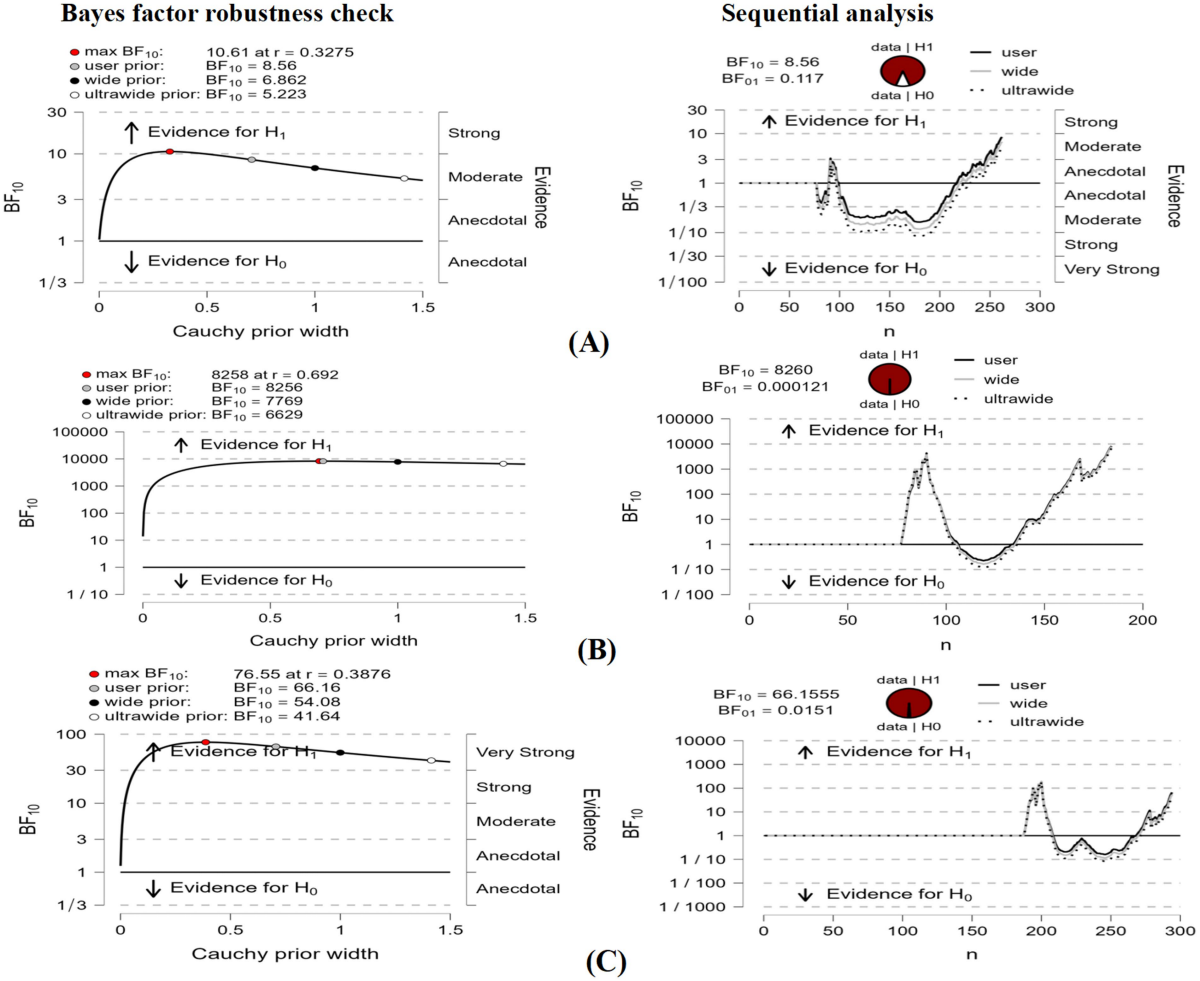
Bayes factor robustness check and sequential analysis.

Between-group differences were observed between low mindfulness and medium mindfulness (BF10 = 8.56), between low mindfulness and high mindfulness (BF10 = 8,260), and between medium mindfulness and high mindfulness (BF10 = 66.16). Figure 3 provides other details.

### Mediating role of resilience based on latent profile analysis

**Figure 4:**
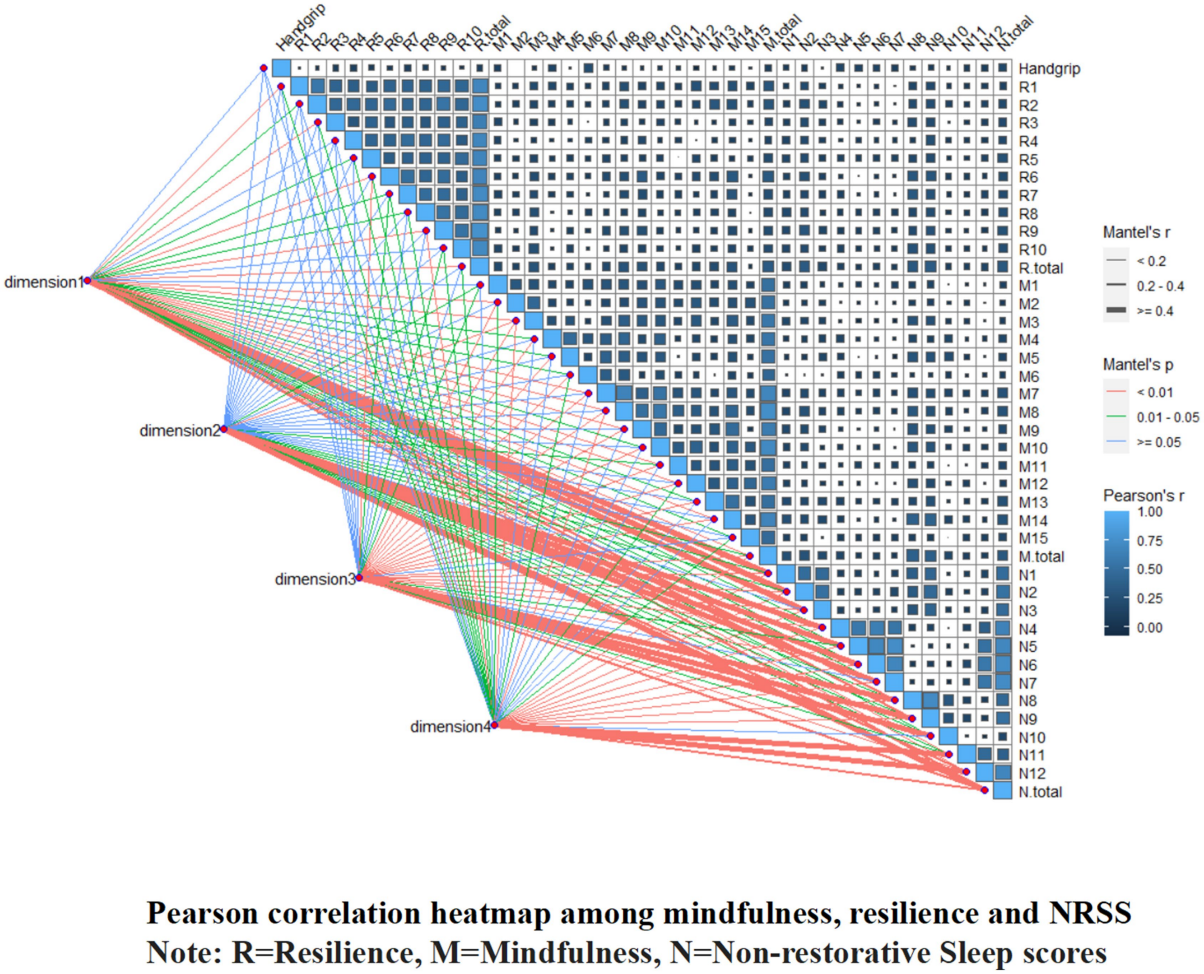
Pearson correlation heatmap among mindfulness, resilience and NRSS.

Significant correlations were observed between mindfulness, resilience, NRS, and handgrip. Pearson’s analysis indicated that mindfulness was positively related to NRSS (*r* = 0.322, *p* < 0.001). Resilience was positively associated with NRSS (*r* = 0.311, *p* < 0.001), mindfulness was positively associated with resilience (*r* = 0.332, *p* < 0.001), and handgrip was positively associated with mindfulness (*r* = 0.106, *p* = 0.042). The Pearson correlation heatmap in Figure 4 provides other details.

Based on the results of LPA, explored the differences in mediating effects between subgroups. Considering low mindfulness as a reference, the results of 95% bootstrap confidence intervals of indirect effect (0.39, 1.46), direct effect (1.33, 4.64), and total effect (2.19, 5.52) indicated that the mediating role of resilience between low mindfulness and medium mindfulness was significant. Additionally, the results of 95% bootstrap confidence intervals of indirect effect (0.43, 1.48), direct effect (0.47, 3.56), and total effect (1.35, 4.47) demonstrated that the mediating role of resilience between low mindfulness and high mindfulness was also significant. Table 1 provides other details.

**Table 1:** The mediation effect of resilience on NRSS among freshmen students.

Variables	*β*	*SE*	*t*	*p*	*LLCI*	*ULCI*	*R2*
	Outcome variable: resilience						0.081
Middle mindfulness	0.505	0.125	4.052	<0.001	0.260	0.750	
High mindfulness	0.519	0.116	4.460	<0.001	0.290	0.747	
	Outcome variable: NRSS						
Middle mindfulness	0.440	0.124	3.555	<0.001	0.197	0.683	0.136
High mindfulness	0.297	0.116	2.562	0.011	0.069	0.525	
Resilience	0.254	0.051	5.010	<0.001	0.154	0.354	

	Direct and indirect effect of mindfulness on NRSS					
	Variables	*Effect*	*SE*	*t*	*LLCI*	*ULCI*
Indirect effect	Middle mindfulness	0.128	0.041	2.666	0.056	0.213
	High mindfulness	0.132	0.040	2.222	0.062	0.220
Direct effect	Middle mindfulness	0.440	0.124	3.555	0.197	0.683
	High mindfulness	0.297	0.116	2.562	0.069	0.525
Total direct	Middle mindfulness	0.568	0.125	4.547	0.322	0.814
	High mindfulness	0.429	0.117	3.678	0.200	0.658

NRSS, Non-Restorative Sleep scores.

### Moderating role of handgrip based on latent profile analysis

The moderating role of handgrip between mindfulness and NRS was not significant. Table 2 provides other details.

**Table 2:** The moderation effect of handgrip between mindfulness and NRSS among freshmen students.

Variables	Estimate	SE	*t*	*p*	LLCI	ULCI
	Mediating variable model (Outcome variable: Resilience)					
Middle-Mindfulness	0.505	0.125	4.052	<0.001	0.260	0.750
High-Mindfulness	0.519	0.116	4.460	<0.001	0.290	0.747
	Dependent variable model (Outcome variable: NRSS)					
Middle-Mindfulness	0.019	0.428	0.045	0.964	−0.823	0.862
High-Mindfulness	−0.165	0.390	−0.422	0.674	−0.932	0.603
Resilience	0.240	0.051	4.764	<0.001	0.141	0.340
Handgrip	0.016	0.006	2.525	0.012	0.004	0.028
Middle-Mindfulness × Handgrip	0.015	0.015	0.999	0.319	−0.014	0.044
High-Mindfulness × Handgrip	0.015	0.013	1.109	0.268	−0.011	0.041
Increase R^2^ with interaction	*R^2^*		*F*		*p*	
	0.1603		11.5497		<0.001	

NRSS, Non-Restorative Sleep scores.

### Generalized additive model analysis between mindfulness and non-restorative sleep

**Figure 5:**
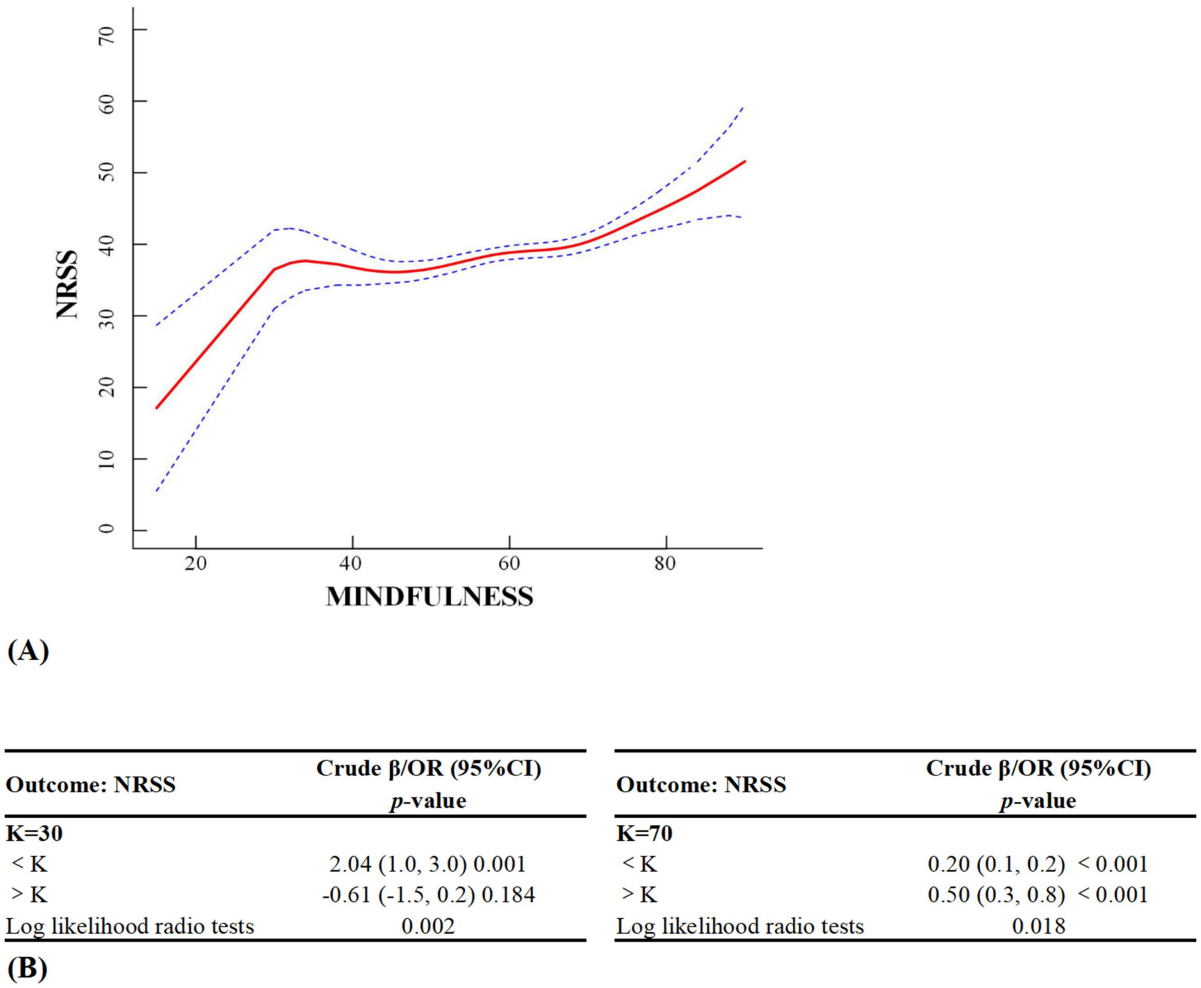
Generalized additive model analysis between mindfulness and NRSS among freshmen students.

All potential variables were controlled in advance. Spline smoothing demonstrated a positive non-linear relationship between mindfulness and NRS, and a significant segmentation effect (i.e., the threshold effect) was observed when the two-piece-wise regression model was used. We found two turning points (*K* = 30, *K* = 70). Before turning Point 30, for every unit increase in mindfulness, NRSS increased by 2.04 units, which was significant (*p* = 0.001). After turning Point 30, for every unit increase in mindfulness, NRSS decreased by 0.61 units, which was not significant (*p* = 0.184). Furthermore, before turning Point 70, for every unit increase in mindfulness, NRSS increased by 0.2 units, which was significant (*p* < 0.001). After turning Point 70, for every unit increase in mindfulness, NRSS increased by 0.5 units, which was significant (*p* < 0.001). Log likelihood ratio tests for Models 1 (*p* = 0.002) and 2 (*p* = 0.018) were significant. Figures 5A,B provides other details.

## Discussion

We found that mindfulness among university freshmen students can be classified into the following three profiles: low mindfulness (20.5%), medium mindfulness (50.3%), and high mindfulness (29.2%). While resilience plays a mediating role between mindfulness and NRS, the moderating role of handgrip between mindfulness and NRS is not significant. Additionally, a positive non-linear relationship exists between mindfulness and NRS.

First, consistent with our first hypothesis, the research sample of mindfulness was classified into three profiles based on a fit index and conceptual support. Specifically, Profile 1 members had a low level of mindfulness and were vulnerable to using maladaptive coping strategies (Miller, 2021), resulting in greater symptoms of insomnia (Schneider et al., 2019). Additionally, heterogeneity in mindfulness among freshmen students indicated different reactions to NRS, which is in line with previous studies (Yalçın et al., 2021). Mindfulness training can buffer the negative impacts of inadequate mindfulness and significantly reduce cortisol secretion to boost well-being, especially in the low mindfulness group (Sousa et al., 2021). Hence, universities should integrate mindfulness and physical condition resources to provide mindfulness-related practice (de Bruin et al., 2015). This can help freshmen students become more positively autonomous than automatic when observing sensations within and outside themselves (Gallego et al., 2014). Furthermore, the results revealed no differences in the variables concerning gender, parental marital status, major, and whether parents live separately. Previous researches indicted that female students have worse sleep quality and more severe physical states and academic stress than male students (Zhou et al., 2022), resulting in worsened NRS symptoms (Kang et al., 2002). However, the imbalance of samples in our study is not sufficient to support this conclusion. Thus, it is necessary to include universities that provide emotional or instrumental support (Eisenbarth, 2019) to help freshmen students relieve their pressure and establish healthy physical conditions.

Second, consistent with our second hypothesis, resilience mediated the relationship between mindfulness and NRS, which is in line with previous findings (Bharti et al., 2023; Niroomandi et al., 2020; Pérez-Aranda et al., 2021). Theoretical research emphasizes that mindfulness has the attribute of consciousness, which can facilitate perseverance traits of resilience by decreasing amygdala activation (Kral et al., 2018). The amygdala has a neural correlation with negative emotions (Hamann et al., 2002), which implies that mindfulness can indirectly suppress the occurrence of poor physical conditions. Conversely, when the amygdala is over-activated, external signs after somatization manifest as insomnia symptoms (Baglioni et al., 2014). To improve NRS, the Internet (Herrero et al., 2019) is a suitable medium to deliver mental interventions, which can enhance resilience and coping strategies in the low mindfulness group, thereby reducing NRS. Furthermore, our study found the indirect effect of resilience between mindfulness and NRS, thereby providing a new method to reduce NRS.

Third, inconsistent with our third hypothesis, handgrip did not play a moderating role between mindfulness and NRS, which may be attributed to the unevenness of the gender in our sample. Additionally, the moderation analysis was performed under the LPA category, which may have caused some deviations in the results. Furthermore, it is necessary to consider handgrip as a physiological indicator and some potential confounding factors such as different testing postures (El-Sais and Mohammad, 2014), environmental changes (Carmelli and Reed, 2000), and testing positions (Boadella et al., 2005) as they may cause significant fluctuations and measurement errors in our outcomes. In addition, our study was limited to a university, and employing limited samples may also contribute to an insignificant moderating effect. Accordingly, future studies should expand our findings to other universities.

Fourth, consistent with our fourth hypothesis, a non-linear relationship was observed between mindfulness and NRS. Based on the generalized additive model, a significant threshold effect was recognized in low mindfulness and high mindfulness groups, which indicates that mindfulness-mediated interventions should be flexible. A prime goal of low mindfulness is to achieve bottom-up emotion regulation with long-term practice (Chiesa et al., 2013), whereas high mindfulness has better cognitive stability regarding exposure to stressful situations (Guendelman et al., 2017), and can use self-adjustment to cope with anxious conditions (Sousa et al., 2021). Hence, individuals with high mindfulness can project their traits externally to mobilize the positive adjustment of others to achieve group effect.

Good sleep is critical for the mindfulness state of college freshmen. Our study found that the mindfulness status is closely related to the sleep quality. Therefore, college freshmen should have the ability to moderate self-adjustment to elevate the level of mindfulness. Considering the association between sleep status and mindfulness in college freshmen, this study may contribute to clinical nursing practice. In their clinical work, psychiatric nurses can derive certain indications from the results of this study. Psychiatric nurses should develop good sleep habits after experiencing heavy daily workloads. Also, efficient mindfulness training can strengthen self-psychological defenses in some degree. Besides, resilience motivates as a buffer between mindfulness and NRS, it revealed that clinical nurses can mobilize psychological state to reach adaptation reorganization to improve sleep state when they have a bad sleep quality.

### Limitations

The present study has some limitations. First, as this was a cross-sectional study, the findings of our study should be verified further by expanding the study population and using a longitudinal design that can encapsulate present outcomes. Second, the sample was obtained from one university, resulting in selection bias. Thus, the findings should be explained with caution. Third, gender imbalance should be considered a potential confounding factor, and future studies should duplicate these findings separately among male and female students. Fourth, a certain measurement error may have occurred in handgrip. Accordingly, more refined grippers should be used to measure their true value.

## Conclusion

Heterogeneity exists in mindfulness among freshmen students. Resilience plays a mediating role between mindfulness and NRS, whereas handgrip cannot moderate the correlations between mindfulness and NRS. A non-linear relationship was identified between mindfulness and NRS.

## Data Availability

Datasets are available on request: The raw data supporting the conclusions of this article will be made available by the authors, without undue reservation.
